# 

**Published:** 1962-01

**Authors:** 


### Contents

DISEASE OF MAJOR CEREBRAL VESSELS *J. L. G. Thomson* 1

THE FASCIAL FLAP OPERATION FOR INGUINAL HERNIA *Edric Wilson* 7

OPEN CARDIAC SURGERY DURING PROFOUND HYPOTHERMIA *A. J. G. Dickens* 11

TRANQUILLIZERS . . . . . . *R. E. Hemphill* 18

MALIGNANT EPENDYMOMA MASQUERADING AS TUBERCULOUS MENINGITIS . . . . *Clinico-Pathological Conference* 28

OBITUARY . . . . . . . . . 35

NOTES AND NEWS 38

APPOINTMENTS . . . . . . . . 38

NEWS FROM THE SOCIETIES . . . . . . 39

